# A generally applicable validation scheme for the assessment of factors involved in reproducibility and quality of DNA-microarray data

**DOI:** 10.1186/1471-2164-6-77

**Published:** 2005-05-20

**Authors:** Sacha AFT van Hijum, Anne de Jong, Richard JS Baerends, Harma A Karsens, Naomi E Kramer, Rasmus Larsen, Chris D den Hengst, Casper J Albers, Jan Kok, Oscar P Kuipers

**Affiliations:** 1 Department of Molecular Genetics, University of Groningen, Groningen Biomolecular Sciences and Biotechnology Institute, PO Box 14, 9750 AA Haren, the Netherlands; 2 Groningen Bioinformatics Centre, University of Groningen, Groningen Biomolecular Sciences and Biotechnology Institute, PO Box 14, 9750 AA Haren, the Netherlands

## Abstract

**Background:**

In research laboratories using DNA-microarrays, usually a number of researchers perform experiments, each generating possible sources of error. There is a need for a quick and robust method to assess data quality and sources of errors in DNA-microarray experiments. To this end, a novel and cost-effective validation scheme was devised, implemented, and employed.

**Results:**

A number of validation experiments were performed on *Lactococcus lactis *IL1403 amplicon-based DNA-microarrays. Using the validation scheme and ANOVA, the factors contributing to the variance in normalized DNA-microarray data were estimated. Day-to-day as well as experimenter-dependent variances were shown to contribute strongly to the variance, while dye and culturing had a relatively modest contribution to the variance.

**Conclusion:**

Even in cases where 90 % of the data were kept for analysis and the experiments were performed under challenging conditions (e.g. on different days), the CV was at an acceptable 25 %. Clustering experiments showed that trends can be reliably detected also from genes with very low expression levels. The validation scheme thus allows determining conditions that could be improved to yield even higher DNA-microarray data quality.

## Background

The development of DNA-microarray technology has enabled genome-wide expression profiling to become a valuable tool in the investigation of an organisms' gene regulation [1-3]. For our studies on gene regulation in Gram-positive bacteria [4] we use in-house developed DNA-microarrays containing amplified DNA fragments of the annotated genes of *Lactococcus lactis *ssp. *lactis *IL1403 [5], *L. lactis *ssp. *cremoris *MG1363 [6], *Bacillus subtilis *168 [7], *Bacillus cereus *ATCC 14579 [8], and *Streptococcus pneumoniae *TIGR4 [9].

Standardization of every step in the DNA-microarray procedure is crucial to correctly and efficiently perform DNA-microarray experiments, and to obtain reproducible data [10-13]. In the process from manufacturing DNA-microarrays to performing the actual experiments, systematic errors and / or bias in the data are introduced in each of the different steps. The effects of various factors (*e.g. *dye and slide) on the quality of DNA-microarray data have been studied quite extensively albeit for experiments performed with eukaryotic systems [14-20]. In contrast, no data quality determination has yet been performed on DNA-microarray data from experiments with bacterial cultures. Furthermore, the effects of different array batches or the influence of the experimenter on data quality have not been included in the previous mentioned experimental designs. Here, we show that the latter factors are indeed important for optimizing DNA-microarray data quality.

In order to assess the reproducibility of, and factors involved in, DNA-microarray data produced in our laboratory during transcriptome analyses by a number of researchers, a validation experiment was designed and implemented. This validation scheme is routinely applied to validate the DNA-microarrays of the various organisms under study in our group. In addition, it allowed to set a quality standard as well as to assess sources of errors in the expression data.

We discuss a novel validation scheme and assess data quality of a number of validation experiments performed on amplicon-based DNA-microarrays of *L. lactis *IL1403. For any laboratory in which DNA-microarray experiments are performed on a regular basis, the validation scheme will provide at the cost of only a few hybridizations, valuable information on the DNA-microarray data quality. Combining multiple validation experiments allows estimation of the main sources of errors.

## Results

### DNA-microarray quality assessment

**Figure 1:**
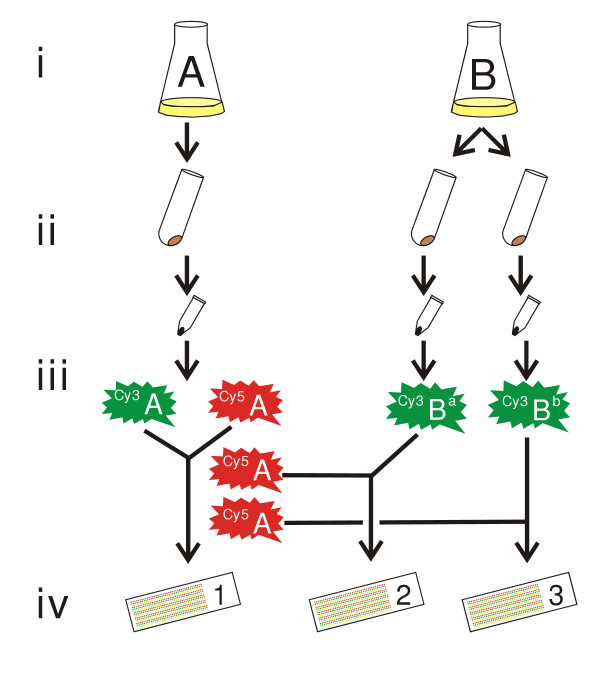
The validation procedure. It consists of 4 steps: (i) cell culturing, (ii) cell pelleting and RNA isolation, (iii) cDNA labeling, and (iv) hybridization, scanning, image- and data analysis.

Six researchers working with *L. lactis *IL1403 slides performed nine validation experiments (see Methods and Figure 1). General statistics on these validation datasets are listed in Table 1. One has to bear in mind that DNA-microarrays with lower signals will yield more noisy data, and thus higher coefficients of variance (CVs). Since these lower signals might also contain valuable information, they are included in the analyses described here.

**Table 1:** General statistics on data obtained from the validation experiments (Figure 1 and supplementary Table S1 [21]).

Validation	Validation slide			5 % low spot filter		40 % low spot filter
						
	1	2	3	CV (%)	Spots (%)	CV (%)

						
A-I	x	x	x	27.3	89.5	17.4
A-I	x			21.6	88.7	13.4
A-I		x		26.1	88.9	17.3
A-I			x	24.6	91.1	16.5
A	x	x	x	16.4	86	12.5
B	x	x	x	13	84.3	9.4
C	x	x	x	14	94.5	8.6
D	x	x	x	16.7	92.1	11.1
E	x	x	x	9.2	90.4	6.7
F	x	x	x	27.5	87.3	20.2
G	x	x	x	16.5	88.5	10.3
H	x	x	x	23.8	91	15.1
I	x	x	x	18.1	92.2	12.4

#### No differentially expressed genes were detected

Differential expression tests were performed for the factors (supplementary Table S1 [21]; *e.g. *spot-pins, experimenters, and validation experiments), but no genes meeting the criteria were observed. No differential expression was expected because the hybridizations were performed with cDNA derived from cells grown under (very) similar conditions. The resulting expression ratios were thus close to 1.

#### CV comparison

The CVs of the validation experiments range from 9 % to 28 % with an average of 17 % and using about 90 % of the spots. The lower CVs of the 40 % low-intensity-spot-filtered data (Table 1) indicate that a significant part of the variance originates from genes with low expression. Slides 2 and 3 of each validation experiment (S2 and S3, respectively) examine biological replicates of independent comparisons between the cultures A and B (Figure 1). Their data quality is thus a "worst case scenario" estimate of the quality to be expected from "real" DNA-microarray experiments as the validation experiments were performed with a large number of differing parameters: (i) different researchers performed the experiments, (ii) on different days, while, lastly, (iii) the cells were harvested in a growth phase in which small changes in culture optical density will result in relatively large differences in expression levels (see below). Table 1 shows, as expected, that data from the pooled slides 1 of all validation experiments (S1) have a smaller average CV (22 %) than those of S2 (26 %) and S3 (25 %). The CV frequency distribution for S1 is shifted towards zero while S2 and S3 have quite similar distributions (supplementary Figure S1 [21]) because of intra-culture differences (B^a ^or B^b^; Figure 1).

#### Detailed comparison of two slides

The two representative validation experiments, i.e. E and H, showed clear differences in data quality (supplementary Table S1 [21]). Box plots of data before the Lowess grid-based normalization show clear spot pin-dependent patterns in average signal levels (supplementary Figure S2 [21]). A non-linear intensity-dependent dye-effect in data from slide E3 (supplementary Figure S2 [21], Graph E2, (i) is evident from the curved Lowess fits. The Lowess curves (one curve fitted for each spotted grid; supplementary Figure S2 [21]) (ii) of slides E3 and H2 are "stacked", indicative of a grid-dependent gradient of ratios. The above-mentioned effects are normalized by using the Lowess grid-based normalization method (supplementary Figure S2 [21], Graph V).

### Gene-dependent fluctuations in ratios and signals

**Figure 2:**
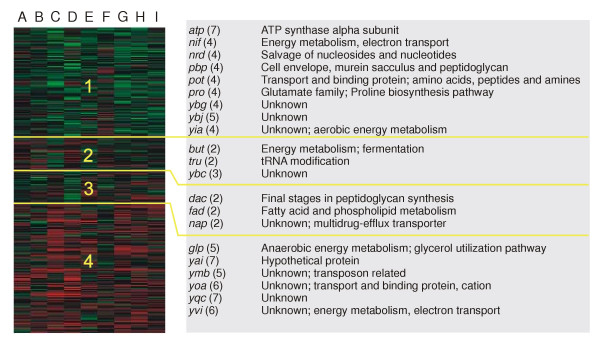
Sammon projection of the clustering of validation data using a self-organizing Kohonen map. Validation experiments (A-I) are shown as well as the clusters (1 – 4; consisting of 761, 230, 227, 886 genes, respectively). Operon names, the number of members, and their (putative) functions are listed to the right of the corresponding clusters. The minimum number of genes in an operon of which all members should be in a certain cluster was determined at a probability of 0.02 or lower for clusters 1 (4 genes), 2 (2 genes), 3 (2 genes), and 4 (5 genes).

Clustering was performed on the SDs of the ratio-data to investigate gene-dependent behavior across the validation experiments (Figure 2). Cluster 1 contains more strongly expressed genes than cluster 4, with clusters 2 and 3 encompassing genes with intermediate expression levels.

#### The clustering results were simplified by grouping genes

A first selection of genes was based on the *L. lactis *IL1403 genome annotation with the underlying assumption that related genes (either by function or because they are part of the same operon) are expected to show similar expression behavior. Only related genes with all members occurring in the same cluster (probability lower than 0.02) were considered.

#### Cell growth-related genes show large fluctuations

Clustering revealed that genes with similar SD fluctuations were involved in (i) amino acid biosynthesis, (ii) energy metabolism, (iii) cell-wall synthesis, and (iv) salvage of nucleosides and nucleotides (Figure 2). Genes showing highest ratio and signal CVs (supplementary Table S2 [21]): (i) are of unknown function, (ii) are (pro) phage-derived, (iii) encode proteins involved in transport of various compounds, or (iv) encode transcriptional regulators.

#### Some genes with low expression show correlated expression fluctuations

**Figure 3:**
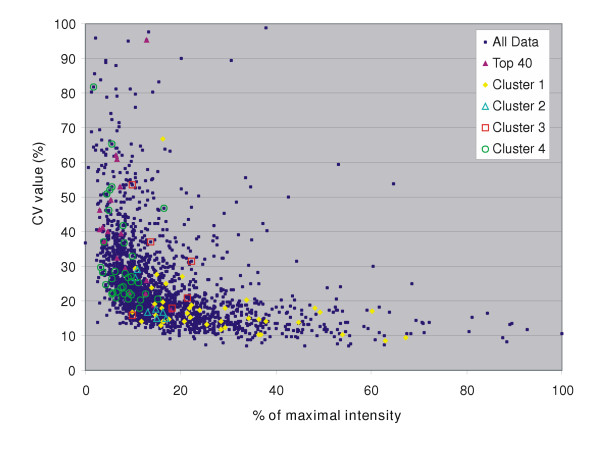
Plot of percentage of maximal intensity versus CV values calculated for the expression levels of genes in the 9 validation datasets (*dark-blue *small squares). *Purple *solid triangles show the top 40 genes with highest variability in ratio and signals (supplementary Table S2 [21]). Functionally related genes showing validation experiment-dependent SDs (Figure 2) are indicated by cluster 1 (solid *yellow *circles), cluster 2 (open *light-blue *triangles), cluster 3 (open *red *squares), and cluster 4 (open *green *circles).

Figure 3 clearly illustrates that (i) the genes with low expression have significantly higher CVs than the highly expressed genes, which is most probably due to their lower signals, and (ii) the related genes (clustered in Figure 3) showing similar expression behavior have average expression levels varying from very low (1.7 % of the maximum intensity) to relatively high (65 % of the maximum intensity). After a close inspection of these (mostly low-intensity) spots, the fluctuations in ratio and / or expression levels did not appear to be correlated to spot quality (data not shown).

### ANOVA

**Figure 4:**
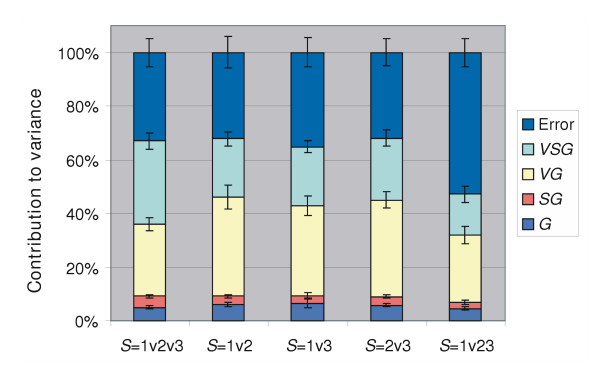
ANOVA results. Each bar represents averages (with error bars signifying the standard deviations for the respective interactions) for 10 random samples of ratio data obtained for the indicated slide combinations (1, 2, and 3; Figure 1). E.g. S = 1v2 indicates a comparison of data from slides 1 with data from slides 2. The interactions (indicated by the colored bars as detailed in the inset) and "Error" (residual variance) amount to 100 % (the total variance present in the data).

A clear correlation between CVs (data quality) and e.g. array batches or experiments could not be determined. For instance, validation experiments H and I were performed on the same DNA microarray batch by the same experimenter, but yielded different CVs. The ANOVA technique allowed estimating the contribution of several sources of errors to the total variance in the DNA-microarray data of all slides (Figure 4; *S *= 1v2v3). The following factors contributed significantly to the total variance: *G *(gene; 5 %; Table 2), *VG *(validation experiment and gene interaction; 27 %), *SG *(slide and gene interaction indicative for dye-effects; 4 %; Table 2), and *VSG *(validation experiment, slides, and gene interactions; 31 %).

**Table 2:** Contribution of sources to the variance estimated for the nine validation experiments (Figure 4) and contribution of individual factors to the *VG *interaction^a^.

Variance source	Contribution to the variance (%)
Gene (*G*)	5.0
Dye (*SG*)	4.2
Gene × Array^b^	7.8
RNA isolation and labeling^c^	1.5
Sampling	7.1
*VG*^d^	26.9

Day × Gene	19.7^e^
Experimenter × Gene	17.3^e^
Array batch × Gene	14.9^e^
Spot pins × Gene	4.5^f^

^a ^The degrees of freedom results in the separate ANOVAs are listed in the supplementary web-site [21].

^b ^Assumed to consist of hybridization effects and signal-to-noise differences per slide.

^c ^Derived from the variance observed between B^a ^and B^b ^cultures (Figure 1).

^d ^Variances that are dependent on the validation experiment performed and due to day-to-day differences, identity of the experimenter, and DNA microarray batch differences.

^e ^Due to overlap in levels, the contribution of these interactions were individually determined.

^f ^A change from 8 to 12 spot-pins used for array spotting coincided with a switch in the RNA isolation method.

#### The *VSG *interaction detailed

In order to distinguish the separate sources of errors in the *VSG *interaction, additional variance analyses were performed with combinations of 2 slides: (i) by omitting slide 1 (S1; containing a self-hybridization) the *VSG *interaction (*S *= 2v3) decreased with 7.8 %; (ii) by omitting slides 2 or 3 (S2 or S3; containing inter-culturing hybridizations) the *VSG *interaction (*S *= 1v2 or *S *= 1v3) decreased with 9.4 % and 9.1 %, respectively; and (iii) the decrease in the *VSG *interactions coincides with an increase of the *VG *interaction. This leads to the conclusion that variances occur on each slide (Gene × Array; Table 2) and may, in part, be due to hybridization effects. Since the variance for a particular slide (7.8 %) is omitted from the variance analyses, the *VSG *interaction will decrease, but the *VG *interaction will increase (the 7.8 % variance was specific for the slide that was omitted from the analyses). This 7.8 % variance is assumed to be the same for each of the three slides. The larger effect of S2 and S3 compared to S1 in the *VSG *interaction is probably caused by the fact that on these slides inter-culture comparisons were performed. Since dye-effects are assumed to be global, it can be concluded that the intra-culturing differences (differences between the B^a ^and B^b ^cultures) account for the 1.6 and 1.3 % larger decrease in the *VSG *interaction (by omitting S2 or S3, respectively). The variance introduced by the B^a ^and B^b ^cultures is quite reproducible (1.3 – 1.6 %) and is caused by RNA isolation and labeling (Table 2).

#### Slide and sampling differences can be determined from *VSG*

The variance of S1 versus the pooled S2 and S3 (*S *= 1v23) in the *VSG *interaction decreased with 16.1 % to 14.9 %, with the variance in the *VG *interaction remaining virtually unchanged. By combining S2 and S3, the Gene × Array interactions occurring specifically on S2 and S3 are pooled. They are, thus, not accommodated in the *VG *interaction, but rather in the residual error. The remaining 14.9 % variance in the *VSG *interaction still contains the Gene × Array interactions for S1 (7.8 %) and sampling differences (7.1 %; Table 2).

#### Day-to-day differences are most prominent in the *VG *interaction

The *VG *interaction contains differences between validation experiments (Figure 4): the DNA microarray batch used (*BG*), day-to-day differences (*AG*), the researcher performing the experiment (*PG*), and spot-pin / RNA isolation method used (*DU*). Due to confounding of these factors, a less efficient estimation of their relative contributions was unavoidable. However, the contributions of *BG*, *PG*, *AG*, *DU *in relation to the *VG *interaction could be determined (Table 2). The day-to-day differences were estimated to have the largest contribution to the variance, followed by experimenter, the DNA microarray batch, and lastly a relatively low contribution of switching the RNA isolation method (coinciding with a change from 8 to 12 spot-pins).

## Discussion

The validation procedure presented here was implemented to provide a standardized method to assess DNA-microarray data quality generated in our laboratory and should be well-suited for use in other laboratories. A workable trade-off between costs, time investment, and data-quality was obtained by using only three DNA-microarray slides for each validation experiment. This scheme is suitable for identifying factors that yield "unreliable" data (*i.e. *data with ratios that deviate from 1 due to, for instance, outliers). In a number of cases, the validation experiment even identified experimenters who did not flag bad spots stringently enough.

Assessment of high-throughput gene expression data quality is a challenging task. A potential problem arises from the fact that many studies do not describe in detail the resulting amount of data on which statistic analyses was based. This information is, however, crucial to determine data-quality. To demonstrate the effect of filtering on data quality, statistics were also calculated for data in which 40 % of the lowest intensity spots were removed (Table 1). These rigorously filtered data do show improved data quality, but at the expense of many measurements that could contain valuable information. The 5 % low-intensity spot filter employed in our study was selected after careful examination of data from various DNA-microarray experiments performed in our laboratory. Some targets with low expression levels allowed grouping genes by function, revealing trends that would have been difficult to discern with more rigorous filtering. A thorough discussion of these results is, however, outside the scope of this study.

The data quality of the validation experiments described in this paper proved to be satisfactory, while at same time a maximum amount of data was preserved. One has to bear in mind that a significant part of the variance in our data is caused by varying factors (e.g. differences in the days on which the experiments were performed; discussed in more detail below). In addition, the quality of the glass surfaces used in this study was lower than that of presently used superamine glass slides (Telechem International Inc.). Together with recently implemented increased stringency of clean-room rules, this will increase data-quality even more. The average CV value for the validation experiments was 26.1 % and 24.6 % for S2 and S3 with use of 90 % of the spots (Table 1). These results are comparable to CVs, ranging from 11 to 23 %, reported for a number of studies using cDNA derived from eukaryotic cell cultures hybridized on various DNA microarray platforms [20,22,23]. For other DNA-microarray experiments performed in our laboratory the data quality is considerably higher (average CVs of under 20 %) stipulating that in effect, the average CV of about 25 % described in this study is an underestimation of the data quality one could obtain.

By mining the data from several validation datasets it was possible to determine which factors contribute to the variance in normalized DNA-microarray data. The following factors were identified (Figure 4 and Table 2): (i) validation experiments (*VG*; 27 %), (ii) sampling (7 %), (iii) Array × Gene (8 %), gene variances (5 %), and dye-effects (4 %). The contributions of RNA isolation and labeling to the variance were quite low (1.5 %; Table 2). Additional variance analyses showed that the day-to-day differences contribute most to the 27 % variance observed for the *VG *interaction, followed by the experimenter, the DNA microarray batch, and lastly a change in the RNA isolation method (coinciding with the use of arrays spotted with 12 instead of 8 spot-pins). The contribution of dye-effects was determined to be only 4 %, which is low compared to the contribution of dye-effects determined for in studies from Chen et al. and Dombrowski et al. [18,24]. The latter study describes the use of a direct labeling kit. In contrast, indirect labeling was used in our study, in which differential hybridization of Cy3 and Cy5-labeled cDNA is anticipated. Direct-labeling adds, next to this differential hybridization, (i) preference of the reverse transcriptase enzyme for the Cy3 label and (ii) prolonged exposure to air and light of the dyes increasing the chance of oxidation and / or bleaching. The main contributing factors identified in this study are in agreement with a number of studies involving cDNA derived from eukaryotic tissue cultures [18,19,25]. In contrast to these studies, we were able to attribute a relatively large contribution of the total variance to specific sources of errors (67 %) because of the efficient design of the validation experiment described here. Since the contributions of day-to-day variation, DNA microarray batch differences, and the experimenter to the variance amounted up to 27 %, it can be concluded that even higher data-quality can be obtained when experiments are performed under identical conditions.

The ANOVA model used does not account for gene-to-gene variances. Additional variance analyses were performed with datasets of which the 10 % most noisy genes (with highest CVs) were omitted. In these experiments, the relative contribution of the various factors identified above remained unchanged (results not shown), indicating that the proposed procedure is robust and that its results are not dependent on a relatively small portion of noisy genes.

In this paper, data from hybridizations with RNA derived from the same experimental conditions were used. To examine whether the probes used on the slides are correct and whether observed gene expression levels are accurate, experiments should be carried out which measure known differentially expressed genes. A number of such studies in which targets were identified by DNA-microarray experiments (*e.g. *on arginine and glucose metabolism and on nisin resistance development), and subsequently verified by alternative techniques (real-time PCR, gene knock-out and / or overexpression studies), have successfully been performed in our laboratory (results not shown).

The validation experiments described in this study were designed to be a "worst case scenario." Data quality proved to be good even though they were obtained at challenging conditions: (i) flask-grown cells, (ii) harvesting in a growth phase in which relatively large changes in gene-expressions occur, and (iii) change of factors (*e.g. *day). These factors represent the conditions under which DNA microarray experiments are performed in our laboratory. Another laboratory could have different factors and levels: e.g. only one researcher that performs the experiments or a different organism under study. Such a laboratory should perform the validation experiments to determine the contribution of the factors that play a role in their particular case. The results of clustering indicate that functionally related genes share specific behaviour across the validation experiments (Figure 3). The significant expression levels and relatively large fluctuations in ratios of the *ybg*, *ybj*, and *yia *gene groups are probably due to biological variations (growth-phase and medium-batch related). Furthermore, one can conclude that data from even genes with very low expression can reveal interesting trends. By preserving the maximum amount of data, one might be able to discern more subtle differences in expression levels of genes with low expression.

## Conclusion

In this paper a novel validation scheme was employed to assess data quality and sources of errors of DNA-microarrays. Even in the case that 90 % of the data were preserved and the experiments were performed at challenging conditions, the coefficient of variance was at an acceptable 25 %. Clustering experiments showed that trends could be detected from genes with very low expression. Using ANOVA, day-to-day as well as experimenter-dependent variances were found to contribute strongly to the variance, while dye and culturing contributions to the variance were relatively modest. The validation scheme thus allows determining conditions that could be used to obtain DNA-microarray data of improved quality.

## Methods

### DNA-microarray experimental procedures

DNA-microarrays were prepared from amplicons of 2108 genes in the genome of *Lactococcus lactis *ssp. *lactis *IL1403 (Genbank accession number NC_002662; its annotation is based on the *B. subtilis *genome, Genbank accession number NC_000964). Primers were designed to amplify unique regions of these genes [26]. Generation of the amplicons, slide spotting, slide treatment after spotting, and slide quality control were performed as described [4] with modifications (see protocols at supplementary web-site [21]). Samples for RNA isolation were taken by rapid sampling of exponentially growing cultures of *L. lactis*. Methods for cell disruption, RNA isolation, RNA quality control, complementary DNA (target) synthesis, indirect labeling, hybridization, and scanning are described in the supplementary web-site [21].

### Validation experiment

The validation experiment (Figure 1) was designed as follows: two independent cultures of *L. lactis *ssp. *lactis *IL1403 were grown at 30°C to an optical density at 600 nm (OD_600_) of 2.0 / cm (corresponding to end-log phase) in standing flasks with 50 mL M17 medium [27] containing 0.5 % glucose (w/v). A 10 mL sample was taken from one of these cultures, while from the other culture two samples of 10 mL were withdrawn. For the validation experiments (supplementary Table S1 [21]), total RNA was extracted using the RNA isolation methods with and without macaloid, for slides made with 12 spot pins and 8 spot pins, respectively. The cDNAs were labeled according to the scheme in Figure 1. The mRNA derived from the A culture was labeled once with Cy3 and three times with the Cy5 dye. The mRNA derived from the B^a ^and B^b ^cultures were both labeled with the Cy3 dye. Finally, the labeled cDNAs were hybridized on *L. lactis *IL1403 DNA-microarrays (Figure 1).

### Data processing

Slide data were processed by using *MicroPreP *[28,29]. (i) spots that were bad (for instance due particles on the slide surface) were manually flagged (for an example see supplementary Figure S3 [21]). These flagged spots were deleted from the datasets because they yield unreliable measurements; (ii) since the spotting buffer contains small random DNA fragments, spots will always have a base signal, particularly in the Cy3 channel, due to autofluorensence of these fragments. The spot backgrounds in each grid for both channels were corrected for this autofluorescense by subtracting the intensity of the weakest spot; (iii) the 5 % or 40 % weakest spots (sum of Cy3 and Cy5 net signals) were deleted. The effect of filtering low-intensity spots from the datasets is demonstrated in supplementary Figure S4 [21]. The 5 % cutoff was determined empirically: the most noisy data is removed from the datasets without removing reliable data; (iv) normalization was performed (the ratios were made comparable across slides) using a grid-based Lowess transformation [30] with f = 0.5 (fraction of genes to use); (v) for both channels the intensities of the "Lowess" fraction of genes were added to yield a total signal, and all intensities were divided by this total signal, yielding scaled, arbitrary expression levels. One has to bear in mind that the scaling procedure affects the signals, but not the ratios. Since the statistical procedures in this paper are based on the ratios, scaling does not affect these analyses; (vi) tables for variance analyses were made. These tables list for each measurement the factors and their levels (see also supplementary Table S1 [21]). For example: spot 1 of slide 1 of validation experiment 1 is gene X (from the gene factor), was obtained by experimenter Y (from the factor experimenter), on day Z (from the factor day).

The scanned images, data, and experimental conditions were stored in the MIAME-compliant Molecular Genetics Information System (MolGenIS) [31].

### Statistical procedures and clustering

The quality of the validation datasets discussed in this paper are presented by coefficient of variance (CV). CVs are calculated by dividing the standard deviation (SD) by the mean ratio of a gene and multiplying by 100 %. The minimum and maximum numbers of measurements for each gene were 13 and 54 (*i.e. *9 validation experiments × 3 slides per validation experiment × 2 technical replicates per slide), respectively. For single validation experiments, CVs and differential expression levels were determined for genes for which at least 4 measurements were available.

Differential expression tests were performed with the Cyber-T implementation of a variant of the t-test [32]. These tests yield for each gene the probability that it has a significantly different ratio than 1. Due to that multiple tests for differential expressions were performed, the false discovery rate (FDR) was determined. The FDR represents the probability that a significant differentially expressed gene is in fact false-positive. FDRs were calculated by (i) ranking the genes by p-value, (ii) multiplying the p-values with the number of tests performed (similar to Bonferroni correction), and (iii) dividing by the number of genes with lower p-values. Genes were considered differentially expressed at both p < 0.01 and FDR < 0.01.

The SDs of log (base 2)-transformed ratios were used for clustering purposes. The clustering technique groups genes which SDs are similar across the validation experiments. The values of SDs for genes with less than four measurements were interpolated by using the K-nearest two neighbours approach using *Engene *[33]: only four genes which lacked the first or last SD had to be omitted. For each gene, SDs were centered after which clustering was performed using the Kohonen self-organizing map (SOM) algorithm (2 × 2 matrix) in the *Engene *clustering package.

### ANOVA

The statistical software package SPSS (version 11; SPSS Inc., Chicago, IL) was used to perform variance analyses (ANOVA). ANOVA determines the contributions of factors (e.g. day) and their levels (e.g. an experiment performed on Monday) to the total variance observed in the datasets. Supplementary Table S1 [21] presents factors and their levels used for ANOVA.

#### ANOVA is robust with respect to violations

The assumptions of ANOVA that (i) error variances are equal and (ii) the residuals of the model are normally distributed generally do not hold for DNA microarray data. However, the sole purpose of ANOVA for this paper was to estimate the relative contributions of the various factors, a purpose for which ANOVA is extremely robust. If the error variances are not equal, the estimators for the type III sums of squares of the various factors, although less efficient, are still valid and unbiased [34]. Furthermore, the efficiency reduces most when the ANOVA design is very unbalanced and/or random factors are implemented [35]. In our case, the design is quite balanced and a fixed-factors model is used. The relative sums of squares are used instead of p-values, because the latter might be violated by deviations from the assumptions.

#### A whole-slide model was chosen over a gene-by-gene model

When performing variance analyses on DNA-microarray data, one can either use a whole-slide model or a more complicated model that allows for gene-to-gene differences. Gene-by-gene models can deal better with variances that are gene-dependent (due to differences in gene expression levels). However, as each of the three hybridized slides (Figure 1) contains different combinations of cDNAs derived from the A and B cultures, the gene expression levels are expected to differ from slide-to-slide, rendering the gene-by-gene method less effective than our whole-slide model.

#### Genes were randomly selected for ANOVA

The software could not handle a gene factor of 2108 levels (genes) and additional interactions in model (1). To reduce data dimensions, we chose to randomly select genes instead of other methods (*e.g. *grouping of genes based on clustering or function) because the latter depend on assumptions of which the validity for the datasets are difficult to determine. The selection was repeated 10 times (with 5 % or 105 random genes each time) yielding 1050 genes of which 196 were drawn two or more times. These 854 uniquely selected genes (40.5 % of the total genes) corresponded well to the predicted 40.0 % (calculated by [1 - (((2108-105) / 2108)^10^)]). The sums of squares were averaged for the sources (*i.e. *factors) contributing significantly to the variance (*α *= 0.05).

#### The ANOVA model uses log-transformed ratio data

Attempts to identify the sources of errors and their contributions to the variance based on signal data, proved to be unsuccessful due to large differences in gene expression levels. A similar observation has been made for oligonucleotide-based DNA-microarrays hybridized with liver tissue RNA [17]. For this reason, we used the following ANOVA model:

*r*_*igpbtv *_= *μ *+ *S*_*i *_+ *G*_*g *_+ *A*_*a *_+ *P*_*p *_+ *B*_*b *_+ *T*_*t *_+ *V*_*v *_+ *U*_*u *_+ *(VG)*_*vg *_+ (*SG*)_*ig *_+ *(VSG)*_*vig *_+ *ε*_*igpbtv *_    (1)

where *r*_*igpbtv *_is the log (base 2)-transformed ratio of gene *g*, which is the *t*^th ^replicate spot on slide *i *performed by experimenter *p *on array batch *b *which was spotted with *u *spot pins (either 8 or 12) in validation experiment *v*. *r*_*igpbtv *_is determined by *μ *(the mean ratio across all the factors) and the global factors slide (*S*), experimenter (*P*), array batch (*B*), day (*A*), the validation experiment (*V*), replicate spot (*T*; 1 or 2), the number of spot pins used (*U*), and a residual error (*ε*_*igpbtv*_). Dye-effects are assumed to be in the *SG *interaction: they are global although the relative contributions of slides 1 – 3 might differ since only slide 1 contains a self-hybridization. The *VSG *interaction contains variances due to hybridization and sampling.

#### Some factors are confounded

Due to the fact that in our DNA-microarray laboratory validation experiments are only performed when necessary (*i.e. *to introduce a new scientist (experimenter) in the laboratory) confounding of some factors could not be avoided. Therefore, variance analyses were performed by employing the validation experiment (*VG*) interaction which incorporates: experimenter (*PG*), array batch (*BG*), day (*AG*), and the number of spot pins, coinciding with a change in RNA isolation method (*GU*).

## Authors' contributions

SvH, AdJ, RB, JK, and OK have designed the validation scheme. SvH, HK, NK, RL, and CdH have conducted the DNA-microarray experiments. SvH, AdJ, and CA analyzed the data and generated the figures. SvH, RB, AdJ, and CA drafted the paper. CA provided guidance with the statistical analysis. JK and OK critically read, revised and approved the final manuscript. All authors read and approved the final manuscript.
